# Analysis of streaming potential flow and electroviscous effect in a shear-driven charged slit microchannel

**DOI:** 10.1038/s41598-020-75531-6

**Published:** 2020-10-27

**Authors:** Adham Riad, Behnam Khorshidi, Mohtada Sadrzadeh

**Affiliations:** Department of Mechanical Engineering, 10-367 Donadeo Innovation Center for Engineering, University of Alberta, Edmonton, AB T6G 1H9 Canada

**Keywords:** Engineering, Mathematics and computing, Physics

## Abstract

Investigating the flow behavior in microfluidic systems has become of interest due to the need for precise control of the mass and momentum transport in microfluidic devices. In multilayered-flows, precise control of the flow behavior requires a more thorough understanding as it depends on multiple parameters. The following paper proposes a microfluidic system consisting of an aqueous solution between a moving plate and a stationary wall, where the moving plate mimics a charged oil–water interface. Analytical expressions are derived by solving the nonlinear Poisson–Boltzmann equation along with the simplified Navier–Stokes equation to describe the electrokinetic effects on the shear-driven flow of the aqueous electrolyte solution. The Debye–Huckel approximation is not employed in the derivation extending its compatibility to high interfacial zeta potential. Additionally, a numerical model is developed to predict the streaming potential flow created due to the shear-driven motion of the charged upper wall along with its associated electric double layer effect. The model utilizes the extended Nernst–Planck equations instead of the linearized Poisson–Boltzmann equation to accurately predict the axial variation in ion concentration along the microchannel. Results show that the interfacial zeta potential of the moving interface greatly impacts the velocity profile of the flow and can reverse its overall direction. The numerical results are validated by the analytical expressions, where both models predicted that flow could reverse its overall direction when the interfacial zeta potential of the oil–water is above a certain threshold value. Finally, this paper describes the electroviscous effect as well as the transient development of electrokinetic effects within the microchannel.

## Introduction

The popularity of lab-on-chip devices has been advancing owing to their decisive influence in a wide range of applications such as medical diagnosis sensors^1,2^, DNA analysis^3–5^, cell sorting^6,7^, surface characterizations^8–10^, and drug delivery^11,12^. Understanding the electrokinetic phenomena in the confined channels of the lab-on-chip devices is essential for the development of accurate and reliable instruments with faster analysis and processing times. Streaming potential flow is an electrokinetic phenomenon that arises due to the relative motion of the electrolyte with respect to a charged wall in a microfluidic channel^13^. The mobile ions within the electric double layer (EDL) near the charged wall are carried by the fluid and result in a convective current, which is known as streaming current. The accumulated ions at the end side of the channel generate an induced streaming electric potential, which results in a conduction current in the opposite direction to the streaming current. The development of conduction and streaming currents creates different ionic fluxes, which can significantly alter the overall flow field depending on the wall surface charge density and electrolyte concertation.

Streaming potential flow can happen in both single and multiphase flows^14,15^. For a solid–liquid interface, the surface charges on a solid surface are the reason behind the formation of EDL within the liquid^16^. Similarly, a liquid–fluid interface can be electrically charged due to the presence of the dissolved ions or charged colloidal particles^17,18^. For instance, an oil–water interface can act as a charged interface where the EDL often forms on the waterside of the interface due to the presence of dissolved salts. For such cases that the liquid–liquid interface is mobile, the oil layer can be assumed as a moving charged surface for electrokinetic analysis. Gao et al.^19^ have examined the steady-state electrokinetic stratified flow of two immiscible fluids analytically in a rectangular channel. They concluded that the interfacial zeta potential has minimal effects on the velocity profile and the volumetric flow rate. However, their analytical model was based on the Debye Hückel approximation limiting their analysis to only low interfacial zeta potentials. Wang et al.^20^ investigated the electrophoretic phenomenon by applying a DC electric field to an oil droplet in the water where the liquid–fluid interface also has electrostatic charges. The external electric field created an electrophoretic force, which exerted on the mobile interface dragging the charges and caused the entire interface to deform and the bubble to move. They concluded that the velocity of the bubble is highly influenced by the zeta potential of the interface and is unaffected by the viscosity and size of the bubble. Li et al.^21^ studied the electrokinetic flow of an oil Janus droplet in a charged circular microchannel with a mobile negatively charged oil–water interface. They were able to predict the velocity of the charged droplet at a steady state for different values of interfacial zeta potential but failed to provide any insight on the transient development of the bubble kinetics. Sherwood et al.^22^ developed a model to predict the streaming potential caused by the motion of a single and a line of charged bubbles passing through a capillary. They concluded that the streaming potential generated across the microchannel is proportional to the applied pressure, where the coefficient of proportionality depends on whether it is an oil or a gas bubble. However, one major shortcoming of their model is that it cannot be used to predict streaming potentials caused by flow adjacent to oil-wet surfaces. Moreover, oil-wet surfaces play an important role in modified salinity water flooding for enhanced oil recovery. This oil–water solution configuration could provide insights into flow observation anomalies within porous rock structures. The literature suggests that the electric double layer is the most dominant surface interaction for this mechanism, while the zeta potential at both rock/brine and brine/oil interfaces directly affects the volume of trapped oil released from the rock^23,24^. Other pertaining issues with literature in electrokinetic flow are the predominant assumptions of steady-state flow during transport and microchannels having infinite lengths^25–32^. Such assumptions do not allow the understanding of the effect of charges present at the inlet and exit of the microchannel. Moreover, the assumption of an infinite microchannel enables the assumption of only radial change in ion concentration, using the one-dimensional Poisson–Boltzmann equation, and ignores the variation of ion concentration in the axial direction, which can be studied using the extended Nernst–Planck equations^32–36^.

In the present work, we aimed at filling the gaps in literature relating to electrokinetic flow in a multilayer system. We propose a model for multilayer flows in a slit microchannel with a finite length. The proposed system consisted of an aqueous solution between a moving plate and a stationary wall. The moving plate represented the charged oil–water interface. The model was used to study the transient development of streaming potential and the subsequent impact of electrokinetic phenomena on the flow field within the microchannel.

## Problem statement

### Analytical analysis

#### The geometry of charged slit microchannel

**Figure 1:**
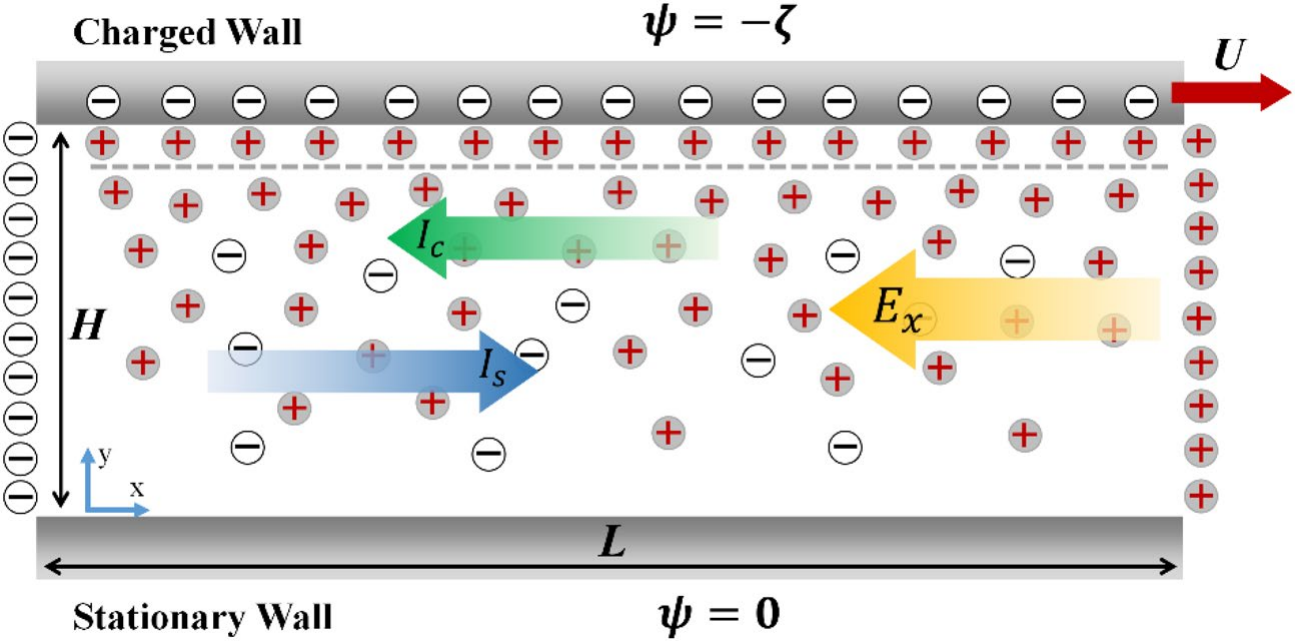
Schematic of the 2D geometry of the charged slit microchannel used for analytical modeling.

Figure 1 illustrates a simplified two-dimensional (2D) geometry of the microchannel used for analytical modeling of the streaming potential flow. The upper wall of the microchannel was charged and moved at a velocity of *U*. The lower wall was considered neutral and stationary. There was one inlet and outlet, carrying electrolyte solution with constant solute concentration. The channel height was (*H*), and the length (*L*) was assumed to be sufficiently large to neglect the entrance and exit effects.

#### Governing transport equations in a charged slit microchannel

The electrokinetic flow in a shear-driven slit microchannel was modeled by Navier–Stokes momentum equations for the velocity field, Poisson’s equation for the electrical potential field, and Nernst–Planck equation for the ion distribution within the channel^37^.

The hydrodynamic flow was modeled using the Navier–Stokes momentum equations with an electrical body force, as shown in Eq. (1):

1 $$\rho \left(\frac{\partial \varvec{u}}{\partial t}+{\varvec{u}}.\nabla \varvec{u}\right)=\mu {\nabla }^{2}\varvec{u}-{\rho }_{f}{\varvec{E}}$$

where *ρ* is the fluid density, ***u*** is the velocity vector, *μ* is the solution viscosity, *ρ*_*f*_ is the free charge density (charge per unit volume of the solution), and ***E*** is the induced electrical field vector which can be represented by the gradient of the electric potential (${\varvec{E}}=-\nabla \Phi$).

For a one-dimensional fully-developed laminar flow field at steady-state, Eq. (1) was simplified to:

2 $$0=\mu \frac{{d}^{2}{u}_{x}}{d{y}^{2}}+{\rho }_{f}\frac{\partial \Phi }{\partial x}$$

The electrical potential distribution was obtained using Poisson’s equation as ${\nabla }^{2}\Phi =-\frac{{\rho }_{f}}{\upvarepsilon }$ where *ε* is the dielectric permittivity of the flowing fluid within the channel.

The electric potential spatial distribution was assumed to be a superposition of the potentials due to the electrical double layer near the charged surface ($\psi (y)$) and the potential developed by the streaming flow ($\varphi \left(x\right)$):

3 $$\Phi (x,y)\equiv \psi (y)+\varphi (x)$$

The assumption of the electrical double potential layer to be independent of the axial position is valid for long microchannels (*L* ≫ *H*) without the end effects^33^. Any deviation from the superposition principle implies that the potential variation over the height of the channel changes at different positions along the x-axis. This is physically impossible in steady flow along a long uniform channel in which the entry and end effects can be neglected^38^. The streaming potential in Eq. (3) was considered as^33,38^:

4 $$\varphi \left(x\right)= {\varphi }_{0}-x{E}_{x}$$

where ${\varphi }_{0}$ is the reference potential at the inlet of the channel ($x=0$) and ${\varphi }_{0}-x{E}_{x}$ is the streaming potential at any axial location in the microchannel due to an axially invariant applied electric field ${E}_{x}$ in the absence of an electric double layer.

The free charge density is related to the concentration of the electrolytic solution by ${\rho}_{f}=\sum z_i en_i$

where *z*_*i*_ is the valence of *ith* ion, *e* is the elementary charge, and *n*_*i*_ is the ionic number concentration of the *ith* ion in the solution.

The total flux of each ionic species (***J***_*i*_) at steady state in the solution is represented by the Nernst–Planck equation as the vector sum of convective, diffusive, and migration fluxes:

5 $${{\varvec{J}}}_{{\varvec{i}}}={n}_{i}{\varvec{u}}-{D}_{i}\nabla {n}_{i}-\frac{{z}_{i}e{n}_{i}{D}_{i}}{{k}_{B}T}\nabla\Phi$$

where *D*_*i*_ is the diffusion coefficient of *ith* species, *k*_*B*_ is the Boltzmann constant, and *T* is the absolute temperature.

Considering the conservation of ions ($\nabla \bullet {{\varvec{J}}}_{{\varvec{i}}}=0$) for the one-dimensional flow, results in Boltzmann distribution for the ionic species as:

6 $${n}_{i}={n}_{{i}_{\infty }}exp\left(-\frac{{z}_{i}e}{{K}_{B}T}\psi \left(y\right)\right)$$

where ${n}_{\infty }$ is the bulk ionic concentration.

Equation (7) was employed to relate the free charge distribution within the channel to the electrical double layer potential distribution for a symmetric (z:z) electrolyte solution as:

7a $${\rho }_{f}=ze{n}_{+}-ze{n}_{-}$$

7b $${\rho }_{f}\left(\mathrm{y}\right)=ez{n}_{\infty }exp\left(-\frac{ze}{{K}_{B}T}\psi \left(y\right)\right)-ze{n}_{\infty }exp\left(\frac{ze}{{K}_{B}T}\psi \left(y\right)\right)$$

7c $${\rho }_{f}(\mathrm{y})=ez{n}_{\infty }(-2\mathrm{sinh}(\frac{\mathrm{z}e}{{K}_{B}T}\psi \left(y\right))$$

Furthermore, introducing the expression for the electric potential and ionic distribution Eq. (6) into the Poisson’s equation gives the nonlinear Poisson–Boltzmann equation as:

8 $$\frac{{d}^{2}\psi \left(y\right)}{d{y}^{2}}=\frac{2ze{n}_{\infty }}{\epsilon }\mathrm{sinh}\left(\frac{ze}{{K}_{B}T}\psi \left(y\right)\right)$$

At steady state, the total current flow per unit width of the channel can be expressed as $I={\int }_{0}^{H}{i}_{x}dy$, where the current density in the x-direction is given by:

9 $${i}_{x}=eu\sum {z}_{i}{n}_{i}-e\sum {D}_{i}{z}_{i}\frac{d{n}_{i}}{dx}+\frac{{e}^{2}}{{K}_{B}T}{E}_{x}\sum {z}_{i}^{2}{D}_{i}{n}_{i}$$

With the assumption of zero ion concentration gradient in the axial direction $\left(\partial {\mathrm{n}}_{\mathrm{i}}/\partial \mathrm{x}=0\right)$, Eq. (9) can be represented by the summation of streaming current (*I*_*S*_) and conduction current (*I*_*C*_) as *I* = *I*_*S*_ + *I*_*C*_.

The streaming current, ${I}_{s}={\int }_{0}^{H}eu\sum {z}_{i}{n}_{i}dy$, is created by the convective transport of the excess ions in the mobile double layer region (diffuse layer) near the charged interface, where the electroneutrality term $\sum {z}_{i}{n}_{i}$ is not zero. The conduction current, ${I}_{C}=\frac{{e}^{2}}{{K}_{B}T}{E}_{x}{\int }_{0}^{H}\sum {z}_{i}^{2}{{D}_{i}n}_{i}dy$, is due to electric conduction caused by the electric field through the liquid along the channel. By employing Poisson’s equation, the streaming current can be represented as

10 $${I}_{S}={\int }_{0}^{H}u\left(-\epsilon \frac{{d}^{2}\psi \left(y\right)}{d{y}^{2}}\right)dy$$

Furthermore, by considering identical diffusion coefficient (*D*_+_ = *D*_*−*_ = *D*) and bulk concentration (${n}_{+\infty }={n}_{-\infty }={n}_{\infty }$) for the positive and negative ions, the conduction current can be expressed as:

11 $${I}_{C}={\sigma }^{\infty }{E}_{x}{\int }_{0}^{H}\mathrm{cosh}\left(\frac{ze}{{K}_{B}T}\psi \left(y\right)\right)dy$$

where ${\sigma }^{\infty }$ is a constant as ${\sigma }^{\infty }=2\frac{{z}^{2}{e}^{2}D}{{K}_{B}T}{n}_{\infty }$.

Table 1 present the list of parameters used in the analytical and numerical simulation. The fluid properties, including were assumed constant and uniform across the microchannel. This assumption is valid for the dilute electrolyte solution, which is typically the case in most microfluidic devices.

**Table 1:** Parameter values used in the present work.

Parameters	Symbol	Unit	Value
Solvent permittivity	$\epsilon$	${\mathrm{C}}^{2}/{\mathrm{Nm}}^{2}$	$78.54\times 8.854\times {10}^{-12}$
Moving wall potential	$\xi$	$\mathrm{mV}$	$-25 \mathrm{to}-250$
Dimensionless channel gap	$\kappa H$	–	$10 \mathrm{to} 100$
Ion valence	${z}_{i}$	–	$1$
Ion diffusivity	$D$	${\mathrm{m}}^{2}/\mathrm{s}$	$1\times {10}^{-9}$
Temperature	$T$	$\mathrm{K}$	$298$
Fluid viscosity	$\mu$	$\mathrm{N s}/{\mathrm{m}}^{2}$	$0.001$
The magnitude of the electron charge	$e$	$\mathrm{C}$	$1.602\times {10}^{-19}$
Boltzmann constant	${K}_{B}$	$\mathrm{J}/\mathrm{K}$	$1.381\times {10}^{-23}$
Moving wall velocity	U	${\mathrm{m/s}}$	$0.001$

### Numerical analysis

#### Geometry and governing equations in numerical simulation

**Figure 2:**
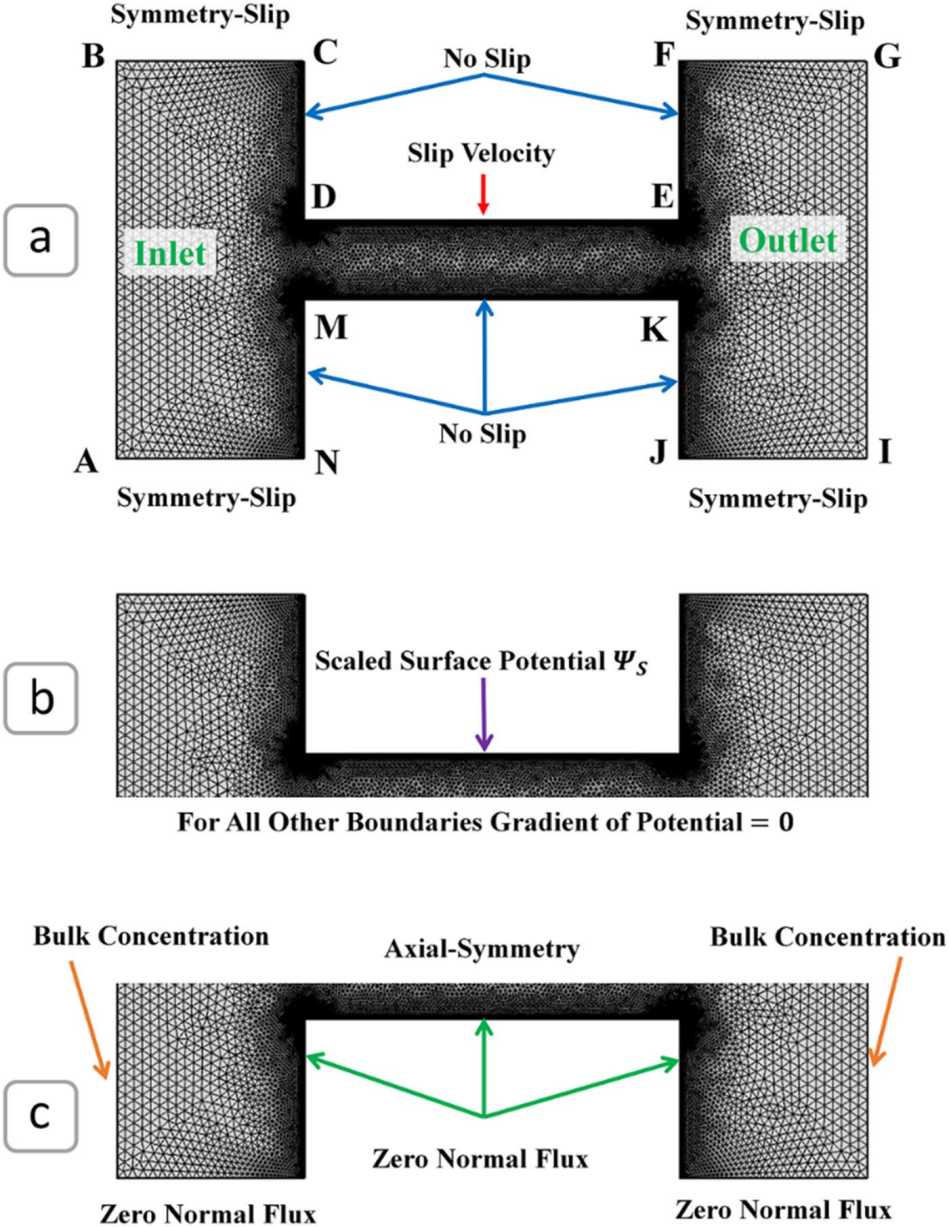
Meshing and boundary conditions for (**a**) momentum equation, (**b**) Poisson’s equation, and (**c**) Nernst–Planck equations.

The numerical model was introduced by Mansouri et al. for pressure-driven flows and was adapted for shear driven flows in our study^39,40^. The simulation geometry consisted of two reservoirs, which were connected by a slit microchannel with length L and height H (Fig. 2). The channel had a stationary electroneutral bottom wall and a charged moving top wall. The numerical simulation was conducted using a similar set of governing equations to the analytical model. The governing equations were non-dimensionalized using the parameters listed in Table 2 where *k* is the inverse of Debye length^41^. For a symmetric binary electrolyte such as an aqueous solution of sodium chloride, the Debye length can be calculated by ${\kappa}^{-1}= {\left(\frac{\epsilon {K}_{B}T}{2{e}^{2}{z}^{2}{n}_{\infty }}\right)}^{1/2}$.

**Table 2:** Non-dimensionalized parameters of the governing equations along with the scaling equations.

Parameter	Non-dimensional form	Relation equation
Axial coordinate	$\overline{x}$	$\kappa x$
Vertical coordinate	$\overline{y}$	$\kappa y$
Time	$\tau$	${\kappa }^{2}Dt$
Gradient	$\overline{\nabla }$	${\upkappa }^{-1}\nabla$
Pressure	$\overline{p}$	$\frac{{z}^{2}{e}^{2}}{\epsilon {\kappa }^{2}{K}_{b}^{2}{T}^{2}}p$
Velocity	$\overline{\mathbf{u}}$	$\frac{1}{D\kappa }{\varvec{u}}$
Fluid viscosity	$\overline{\mu }$	$\frac{{z}^{2}{e}^{2}D}{\epsilon {K}_{b}^{2}{T}^{2}}\mu$
Fluid density	$\stackrel{-}{\rho }$	$\frac{{z}^{2}{e}^{2}{D}^{2}}{\epsilon {K}_{b}^{2}{T}^{2}}\rho$
Ion concentration	${\overline{n}}_{p},{\overline{n}}_{n}$	$\frac{{n}_{p}}{{{n}_{\infty }}},\frac{{n}_{n}}{{n}_{\infty }}$
Free charge density	$\stackrel{-}{{\rho }_{f}}$	$\frac{1}{ze{n}_{\infty }}{\rho }_{f}$
Electric potential	${\overline{\psi }}_{d}$	$\frac{ze}{{K}_{b}T}\psi$

By substituting the parameters of Table 2 into Eq. (1), and eliminating the convective term ($\bf u\bullet \nabla \mathrm{\bf u}$) for creeping flows such as flow in microchannels, the non-dimensional form of momentum equation can be written as:

12 $$\stackrel{-}{\rho } \frac{\partial \overline{\mathrm{\bf u}}}{\partial \tau }=-\overline{\nabla } \overline{p}+\overline{\mu } {\overline{\nabla }}^{ 2}\overline{\mathrm{\bf u}}-0.5 \left({\overline{n}}_{p}-{\overline{n}}_{p}\right) \overline{\nabla } {\overline{\psi }}_{d}$$

where ${\overline{n}}_{p}$ and ${\overline{n}}_{n}$ are the scaled concentrations of the co-ions and counterions for a symmetric binary electrolyte solution, respectively.

The non-dimensional form of Poisson’s equation using Table 2 becomes:

13 $${\overline{\nabla }}^{2} {\overline{\psi }}_{d}=-0.5 \left({\overline{n}}_{p}-{\overline{n}}_{p}\right)$$

Finally, the non-dimensional Nernst–Planck equations for the transport of positive and negative ions are presented by Eqs. (14) and (15), respectively:

14 $$\frac{\partial {\overline{n}}_{p}}{\partial \tau }=-\overline{\nabla } . ( {\overline{n}}_{p} \overline{\mathbf{\bf u}}-\overline{\nabla } {\overline{n}}_{p}-{ \overline{n}}_{p} \overline{\nabla } {\overline{\psi }}_{d})$$

15 $$\frac{\partial {\overline{n}}_{n}}{\partial \tau }=-\overline{\nabla } . ( {\overline{n}}_{n}\boldsymbol{ }\overline{\mathbf{\bf u}}-\overline{\nabla } {\overline{n}}_{n}+{ \overline{n}}_{n} \overline{\nabla } {\overline{\psi }}_{d})$$

The time-dependent terms in the governing transport equations imply the coupled transient dependence of the physical models to each other. The simulation started initially from a quiescent point at zero flow conditions, and then the transient behavior of the system was traced until it reached a steady-state flow.

#### Boundary and initial conditions of the numerical simulation

Figure 2 illustrates the boundary conditions of the simulation domain. For the momentum equations, the inlet and outlet boundary conditions (A–B and G–I) were set to identical pressure to eliminate the pressure gradient through the channel. The flow is thus primarily caused by the slip velocity boundary condition applied to the top wall of the channel (D–E). This slip velocity boundary condition is defined as the relative velocity in the tangential direction between the top wall (D–E) and the initial frame of reference of the numerical model $\overline{\mathbf{u}}\bullet \overline{\mathbf{t}}={\overline{\mathrm{U}}}_{\mathrm{Wall}}$, where $\overline{\mathbf{t}}=\left({n}_{y}, -{n}_{x}\right)$ for our 2-dimensional case. The upper walls of the reservoir (B–C, F–G, A–N, and J–I) were considered as slip boundaries to mimic the behavior of a semi-infinite reservoir^40^. For the other boundaries, the Dirichlet no-slip boundary condition was employed to emulate the behavior of a stationary wall. It is worth mentioning the slip length between the oil and the water phases is assumed to be negligible in our model. In microfluidic systems, the typical slip lengths reported experimentally range from molecular dimensions to several nanometers^42^. Therefore, the impact of slip in microchannels, like our proposed system, can be negligible. For the Poisson equation, a scaled surface potential was assigned to the top wall (D–E) of the microchannel (Fig. 2b). The reference potential of zero was set at the flow inlet (A–B) to represent the charge neutrality far field condition. For the other boundaries, zero potential gradient (Neumann boundary condition) was applied to present a far-field condition with no variation in the potential distribution. Finally, for the Nernst–Planck equations, the inlet and outlet boundaries were assigned as symmetric neutral electrolytic solutions with a scaled ion concentration of one to represent the bulk ion concentration (Fig. 2c). All other boundaries were assigned as zero normal flux conditions(Neumann boundary condition) to be impermeable to any ions transport. The steady-state solution of the Poisson and Nernst–Planck equations, along with the no-flow quiescent state, were used as the initial conditions for the transient flow analysis. The computational geometry was created for *κH* of 10, which translates to the height of the channel being ten times bigger than the Debye length. The scaled length of the microchannel, *κL*, was set to 50, and the scaled lengths of the inlet and outlet reservoirs (A–N and J–I) were assigned a value of 25.

#### Numerical solution methodology

The nonlinear partial differential equations were solved using a commercial fully coupled finite element solver incorporated in COMSOL Multiphysics (V 5.3)^43^. The methodology involved a segregated solution method in which both the steady-state solutions for the Poisson and Nernst–Planck equations were obtained for the no-flow case in order to get the quiescent electric potential and ion distributions^44^. Then, these distributions were inputted as the initial values for the momentum equations in order to solve for the velocity domain. In the initial phase of streaming potential, several dynamic processes, including the capacitive charging of the electric double layer, take place. The basis for using this segregated approach in our methodology is to separate the effect of these initial dynamic processes from the development of the flow field. All the four governing equations, i.e., Eqs. (15–18), were solved together in a coupled manner in order to capture the axial ion displacement within the channel, which would ultimately lead to the development of the induced streaming potential. In the transient simulations, the steady-state condition was achieved when the potential difference between the two reservoirs for two consecutive time steps became almost the same within a predefined tolerance^41^. The mesh map was systematically refined until the solution became mesh independent at around 15,000 elements. The time needed for the simulations was within hours, highlighting the efficiency of the chosen computational domain. The mesh generation was performed using quadratic triangular elements with a finer mesh near the upper wall. This was the region where the largest gradient in the electrical potential and the velocity was expected^44^.

## Results and discussion

### Analytical analysis of shear-driven streaming potential flow

#### EDL potential distribution within the channel

The electrical potential distribution within the electric double layer (EDL), in the straight microchannel (Fig. 1), can be obtained using Eq. (16):

16 $$\Psi \left(y\right)=4 arctanh\left(\mathrm{tanh}\left({\Psi }_{s}/4\right)exp\left(-\kappa (H-y)\right)\right)$$

where $\Psi \left(y\right)$ and ${\Psi }_{s}$ are dimensionless electrical potentials defined as $\Psi \left(y\right)=ze/{K}_{B}T\psi \left(y\right)$ and ${\Psi }_{s}=ze/{K}_{B}T\xi$.

**Figure 3:**
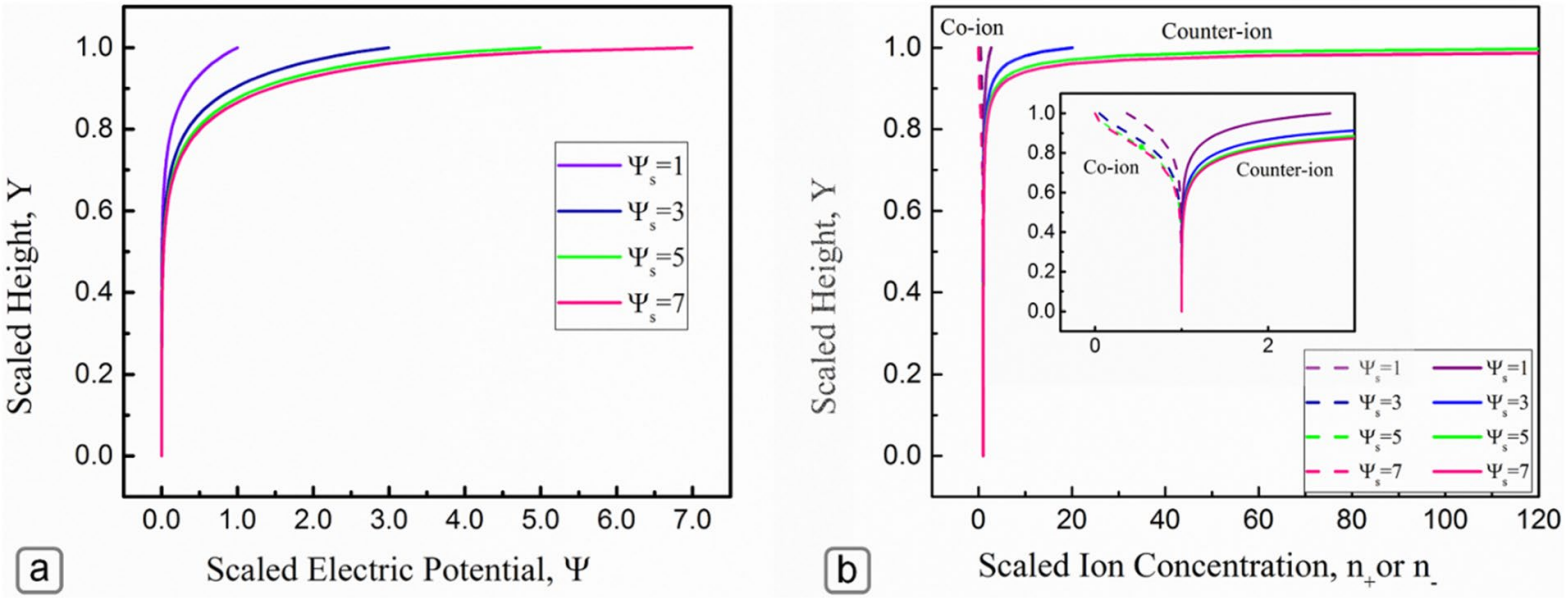
(**a**) Distribution electric potential, and (**b**) distribution of co-ions and counter ions within the channel where the dashed lines are for the co-ions while the solid lines are for the counterions (*κH* = 10).

Figure 3 illustrates the EDL potential distribution as well as the distribution of ions within the microchannel for different surface zeta potentials of the upper wall. Based on Fig. 3a, for low surface zeta potentials, the potential distribution within the channel decayed quickly to zero suggesting the formation of narrow EDL near the upper wall. In contrast, at higher surface potentials (${\Psi }_{s}=9)$, the EDL extended further to about mid-height of the channel, which indicated the formation of a thicker EDL by increasing the zeta potential of the upper wall. Figure 3b demonstrates similar behavior for the distribution of ion concentrations over the channel height$.$ At low surface potential (${\Psi }_{s}=1$), the spatial distribution of the co-ions and counterions were slightly affected by the presence of the charged wall. However, as the surface zeta potential of the upper wall increased, more concentrations of counter ions were attracted to the charged surface and resulted in the formation of thicker EDL near the upper wall.

#### Induced electric field

An analytical expression for the induced electric field *E*_*x*_ was derived by considering a zero net current within the channel at a steady state. Starting with conduction current expression, substituting Eq. (16) into Eq. (11) provided the relation between the conduction current and the potential distribution as:

17 $${I}_{C}={\sigma }^{\infty }{E}_{x}H{\int }_{0}^{H}\mathrm{cosh}\left(4 arctanh\left(\mathrm{tanh}\left({\Psi }_{s}/4\right)exp\left(-\kappa y\right)\right)\right)dy={\sigma }_{\infty }{E}_{x}H {F}_{cs}$$

where *F*_*CS*_ is the parameters under the integral sign and presents the non-electroneutrality of the solution ($\sum {z}_{i}^{2}{n}_{i}\ne 0$) due to the formation of the electrical double layer near the charged wall^33^.

**Figure 4:**
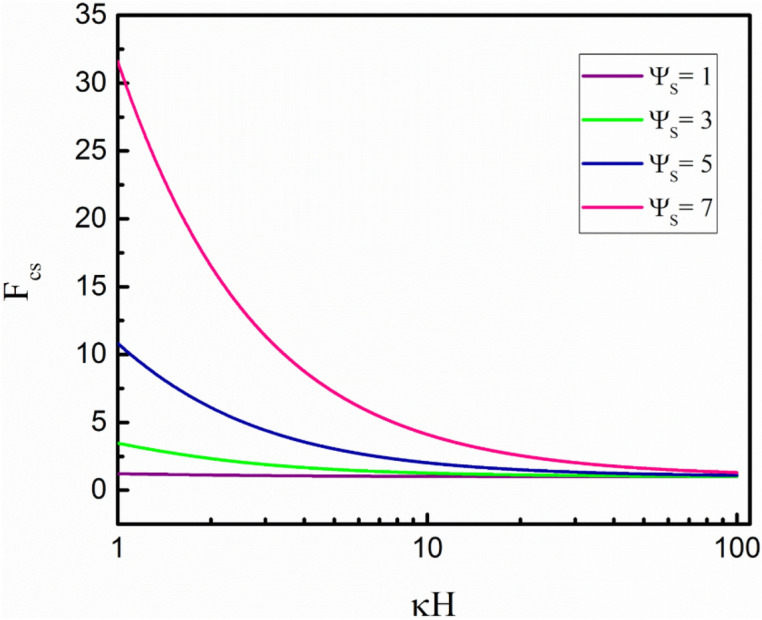
Variation of the *F*_*CS*_ with surface potential for different *κH*.

Figure 4 illustrates the variation of *F*_*CS*_ versus *κH* at different non-dimensional wall surface potentials (Ψ_*S*_). By increasing the Ψ_*S*_, the *F*_*CS*_ values increase due to the stronger redistribution of ions near the charged wall, leading to more deviation from the electroneutral state within the fluid layer. Furthermore, the *F*_*CS*_ values decrease as *κH* increases and approach unity at a very large *κH*. This observation can be interpreted that when the EDL thickness decreases (*κH* increases), the nonuniform distribution of ions will be limited to a smaller scale near the charged wall, and thus, more electroneutrality will be achieved in the liquid phase.

The induced electric field (*E*_*x*_) due to streaming potential flow can be formulated by assuming that at steady-state condition, the streaming and conduction currents will be equal in the opposite direction, resulting in a zero net electric current through the liquid. Setting the total electrical current to zero, $I={I}_{C}+{I}_{S}=0$, and solving for *E*_*x*_ gives the strength of the induced electric field as:

18 $${E}_{x}=-\mu U\Omega$$

where

19 $$\Omega =\frac{{C}_{1}\kappa H+\xi }{\mu D{\left(\kappa H\right)}^{2}{F}_{cs}+{C}_{2}\kappa H\epsilon -\epsilon {\xi }^{2}}$$

and the constants C_1_ and C_2_ are

20 $${C}_{1}=-2\left(\frac{{K}_{B}T}{ze}\right)sinh\left({\Psi }_{s}/2\right)$$

21 $${C}_{2}={8\left(\frac{{K}_{B}T}{ze}\right)}^{2}{sinh}^{2}\left({\Psi }_{s}/4\right)$$

**Figure 5:**
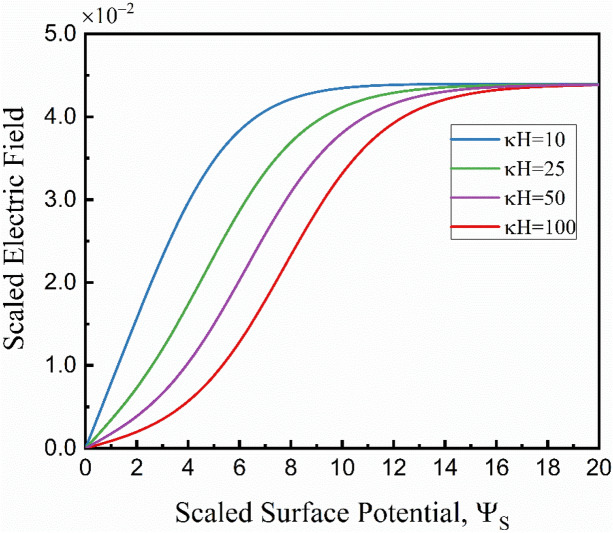
Variation of the induced electric field (*E*_*x*_) vs. non-dimensional surface potential (Ψ_*S*_) at different *κH.*

Figure 5 demonstrates the variation of *E*_*x*_ with respect to Ψ_*S*_ at different *κH*. The magnitude of the induced electric field increases by the increase in surface potential of the upper wall. A higher surface potential increases the ionic concentration within the electric double layer. Therefore, more ions move due to the shear-driven flow and buildup larger concentration at the ends of the channel resulting in higher induced electric field magnitudes. Furthermore, the *E*_*x*_ increases more rapidly at smaller *κH* values. For a diluted solution (small *κH*), the ionic concentration (*n*_*∞*_) is low. Since the conduction current is directly proportional to the ionic concentration, a higher *E*_*x*_ is required for a diluted solution to satisfy the net-zero current at the steady-state compared to a concentrated electrolyte solution (large *κH*).

#### Velocity field and volumetric flow within the microchannel

The scaled local velocity field within the microchannel is presented by Eq. (22), which was derived by employing Poisson’s equation for electric potential, along with the boundary conditions of *u*_*x*_ = *U* at *y* = *H* and *u*_*x*_ = *0* and *y* = *0* for momentum equation in axial direction:

22 $${U}^{*}\left(y\right)= \frac{u_x\left(y\right)}{U}=\left(\frac{y}{H}\right)-\epsilon \Omega \left(\psi (y)-\xi \left(\frac{y}{H}\right)\right)$$

**Figure 6:**
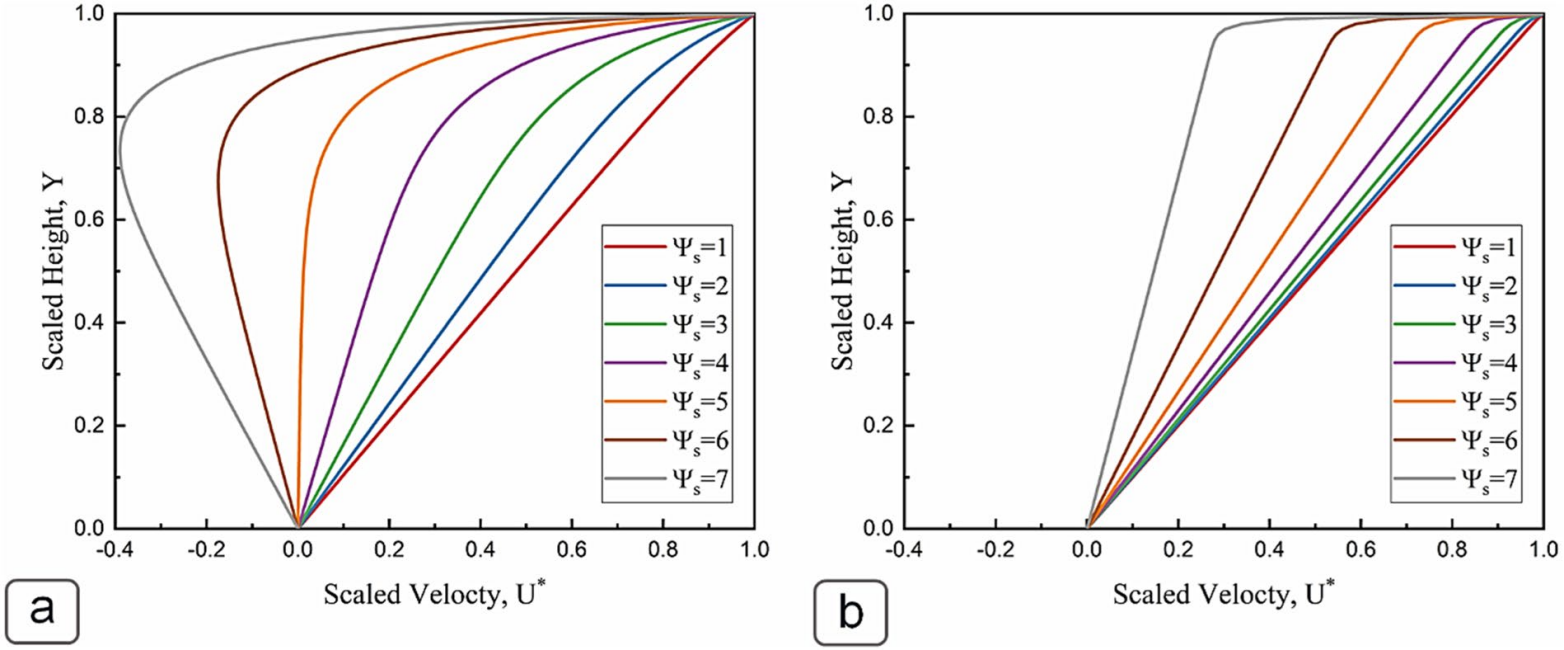
Effect of surface potential (${\Psi }_{s}$) on non-dimensional velocity profile, (**a**) *κH* = 10, (**b**) *κH* = 100.

Equation (22) implies that the velocity field within the channel is a superposition of two components: (1) a linear shear-driven flow, and (2) a streaming potential flow. The variation of scaled local flow velocity at different scaled surface potentials of the upper moving wall is presented in Fig. 6a (for *κH* = 10) and Fig. 6b (for *κH* = 100). Figure 6a shows that for small surface potentials, the velocity profile is almost linear, suggesting the fluid is entirely driven by viscous flow due to the motion of the upper wall, and the reverse streaming potential flow has a negligible effect on the velocity distribution. However, at higher surface potentials, the velocity profile becomes nonlinear, revealing the significant effect of the electrokinetics on the flow field. The shear driven flow is proportional to the slip velocity of the moving wall, while the streaming potential flow is proportional to the surface potential of the moving wall. Therefore, as the surface potential increases, the velocity profile deviates more from the linear profile, due to the buildup of an opposing streaming potential flow. For scaled surface potentials above ${\Psi }_{s}=5$ (~ 125 mV), the streaming potential backflow became sufficiently large to reverse the direction of the net flow velocity at transverse positions near the moving charged wall. Such high zeta potentials are common in enhanced oil recovery applications. Moutray, Leduc Crude, and ST-86-1 oils all exhibit an interfacial zeta potential above 100 mV at 0.01 M NaCl solution and pH values greater than 10^45^. Figure 6b illustrates the impact of surface potential on the velocity profile for a large *κH* value of 100, which is the case for a microchannel with a concentrated electrolyte solution. As EDL is very thin in this case, the mobile counterions in the diffuse layer are very close to the charged wall, and thus the electroneutral bulk phase of the fluid follows the shear-driven flow field.

The volumetric flow can be obtained by integrating the velocity field over the cross-section area of the microchannel as:

23 $$Q= \frac{H}{2}\left[U-\frac{\varepsilon {E}_{x}\zeta }{\mu }\right]+\frac{\varepsilon {E}_{x}\zeta {K}_{b}T}{\mu ze}\left[\frac{4\mathrm{tanh}(\Psi s/4)\left(1-{e}^{-\kappa H}\right)}{\kappa\zeta }+\frac{{4\mathrm{tanh}}^{3}(\frac{{\Psi }_{s}}{4})\left(1-{e}^{-3\kappa H}\right)}{9\kappa\zeta }\right]$$

**Figure 7:**
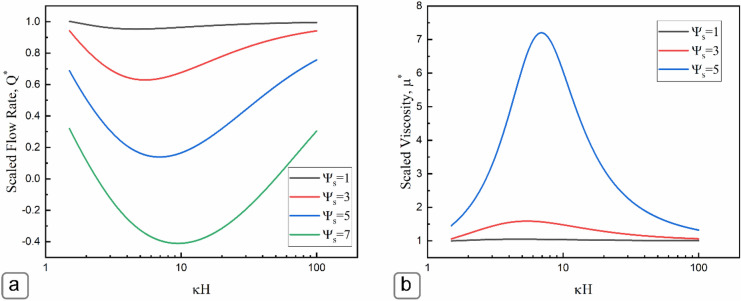
(**a**) A plot of non-dimensional flow rate versus *κH* for different surface potentials; (**b**) plot of normalized viscosity $\frac{{{\varvec{\mu}}}_{{\varvec{a}}}}{{\varvec{\mu}}}$ versus *κH* The flow rate was scaled by the volumetric flow rate in the simple shear driven flow case with the absence of any electrokinetic effect *Q*_*scaling*_ = *UH/2*.

Figure 7a demonstrates the variation of non-dimensional volumetric flow rate with respect to *κH* at different scaled surface potentials. The graph shows that the induced streaming potential reduces the volumetric flow rate, especially for low values of *κH*. The reduction in the volumetric flow rate resembles the flow of a fluid with increased viscosity, which is called the “electroviscous effect”. Figure 7b presents that the non-dimensional viscosity enlarges at low *κH* values with the maximum at *κH* = 5*,* and then reduces at higher *κH* values. As the surface potential increases (Fig. 7a), the electroviscous effect becomes larger and reduces the volumetric flow rate more. For instance, at the surface with zeta potentials of around 75 mV (${\Psi }_{s}=3)$, the electroviscous effect can reduce the flow rate by about 35% at *κH* ~ 5. For very high surface potentials (${\Psi }_{s}=7)$, the “electroviscous effect” dominates over the shear driven flow resulting in negative values for the volumetric flow rate at *κH* ~ 10.

A potential application for the significant electroviscous effect (reduction in apparent viscosity) is microfluidic drug delivery. Most antibody formulations are highly viscous, which poses a significant challenge for the subcutaneous delivery of these highly concentrated protein formulations using microfluidic devices^46–48^. Designing a microfluidic system with high surface potential and the addition of specific additives to control the electrolyte conditions can be used to reduce the viscosity of these fluids to manageable levels. This reduction in viscosity is critical for drug delivery as it allows for more control in dose administration, increased patient compliance and comfort, and reduces the overall drug costs due to its substitution of intravenous infusions.

### Numerical modeling of flow in finite microchannel

#### Validation of the simulation analysis

**Figure 8:**
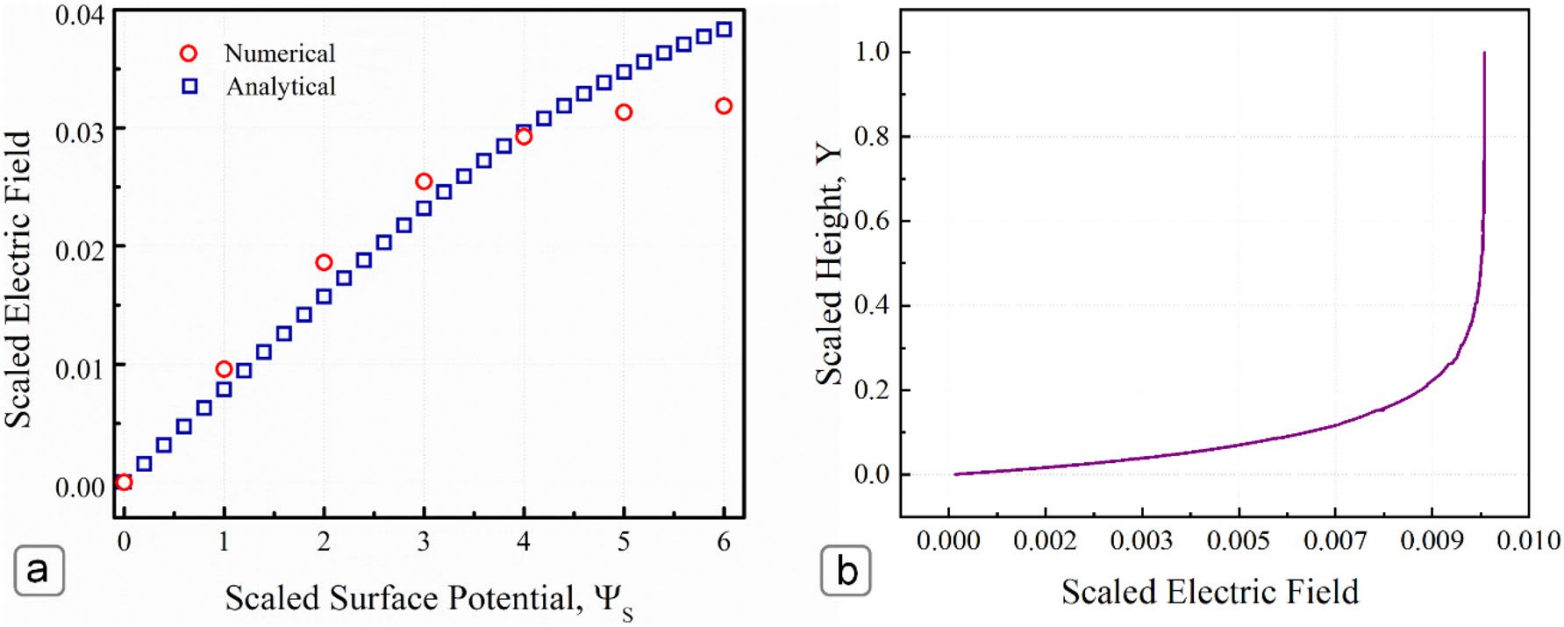
(**a**) Comparison of the numerical and analytical predictions of the induced electric field for different surface potentials, *κH* = 10. (**b**) Scaled electric field distribution, *κH* = 10. For a comparison of the numerical and analytical predictions of the induced electric field for *κH* = 100 (see Supplementary Figure S1).

Figure 8a presents the comparison between the results of the numerical simulation and analytical analysis for the induced electric field in the microchannel at the steady-state condition. The numerically predicted electric field was in good agreement with the analytically derived electric field, especially at low surface potentials. However, for larger surface potentials (Ψ_*S*_) above 4, the numerical simulation predicted a lower magnitude of the induced electric field compared to the analytical solution. The discrepancy can be ascribed to the simplifying assumptions employed in the derivation of the analytical expression. The analytical expression can not capture the non-linearity of the velocity profile associated with high surface potentials (Ψ_*S*_ > 4). This deficiency can be attributed to using the simplified one-dimensional Poisson–Boltzmann equation in the analytical expression rather than solving the Poisson and Nernst–Planck equations simultaneously. This simplification consequently led to incorporating a constant electric field in the transverse direction for the analytical expression while the numerical model predicted a varying electric field, as shown in Fig. 8b. The effect is exaggerated as the induced electric field increases in magnitude. For low surface potentials and in microchannels where L ≫ H, the transverse fluctuations in the electric field are negligible and can be assumed constant. However, for high surface potential and microchannels with comparable length to height ratio, the effect this variation has on the induced electric field and, consequently, the velocity profile inside the channel is significant.

**Figure 9:**
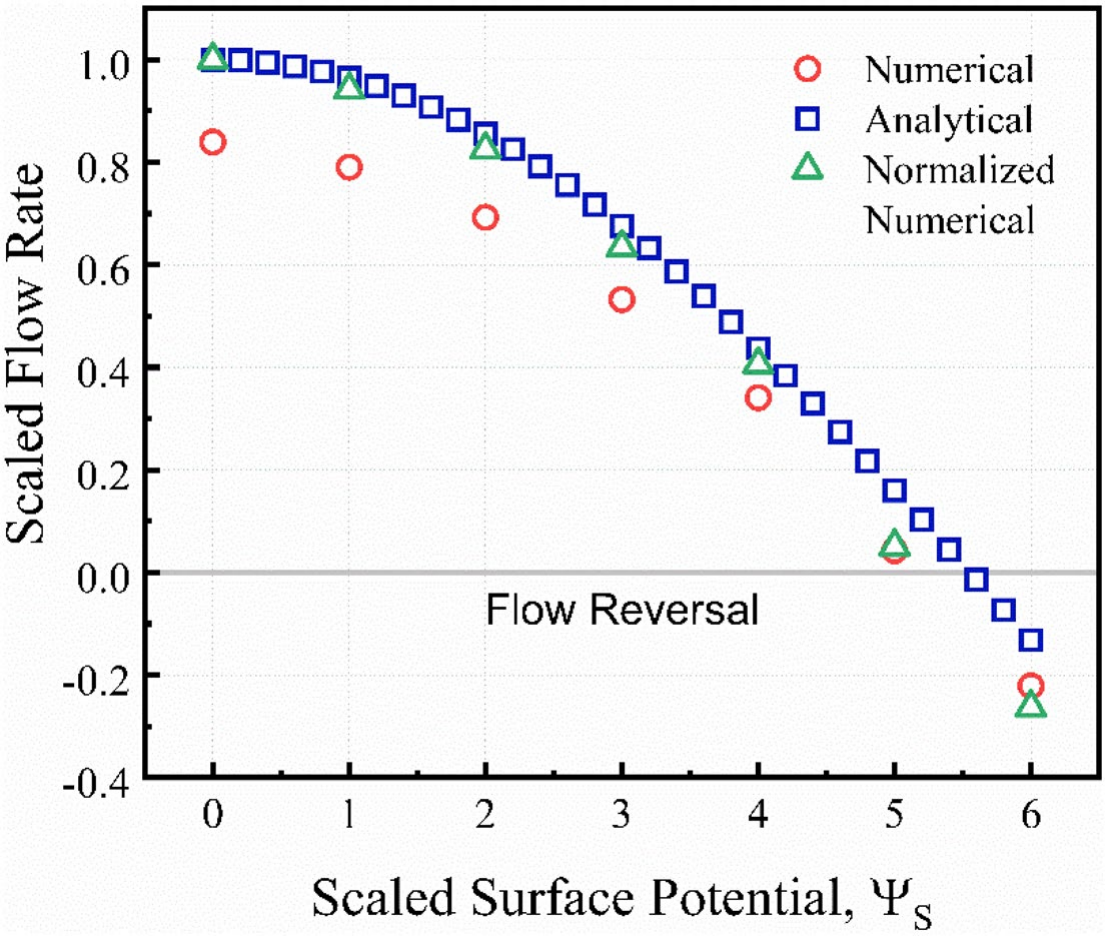
Comparison of the numerical (circles) and analytical predictions (squares) of the scaled flow rates for different surface potentials, *κH* = 10. The normalized numerical flow rates (triangles) is the normalized flow rate by the numerical result at Ψ_*s*_ = 0 (finite microchannel with simple shear driven flow case)*.*

Figure 9 presents a comparison of the numerical simulation with the analytical model for the scaled flow rate and the flow reversal phenomenon. Again, the predictions of the numerical simulation were in good agreement with the analytical expressions. However, there is a reasonable disagreement (16.1%) between the numerical predictions and analytical results for the simple shear driven flow case in the absence of any electrokinetic effects (Ψ_*s*_ = 0). The reason behind this disparity is the infinite length assumption of the analytical model. The analytical model relies on a linear velocity distribution for shear-driven flows. However, this assumption is not valid for microchannels with finite dimensions, leading to the entrance and exit effects for the flow of the electrolyte solution. To investigate the stagnation point (where flow starts to reverse its overall direction), the obtained numerical simulation results were normalized by the flow rate at Ψ_*s*_ = 0 to discard any dimensional effects from our comparison. The comparison of the normalized numerical results shows a better agreement with the analytical expressions. Both the analytical expressions and the numerical model show that the stagnation point occurs for microchannels with a scaled surface potential above ${\Psi }_{s}=5$ (~ 125 mV). The analytical model predicts an exact value of Ψ_*s*_ = 5.56 at the stagnation point.

#### Transient analysis of the streaming potential flow in the microchannel

Model development for transient electrokinetics can provide more insights into the practical development and operation of lab-on-chip devices. For example, the efficiency of electrophoretic-based separation strongly depends on evolution parameters such as the injection rate and the rise time of the flow. Additionally, high-speed electrokinetic pumps were reported to achieve the separation process within the micro-second range^49^. Depending on the position of molecules in the transverse direction, the rise time for this separation process is estimated to be in the order of ${10}^{-9} -{10}^{-5}\mathrm{s}$ (thousands of the diffusion time scale)^50^.

**Figure 10:**
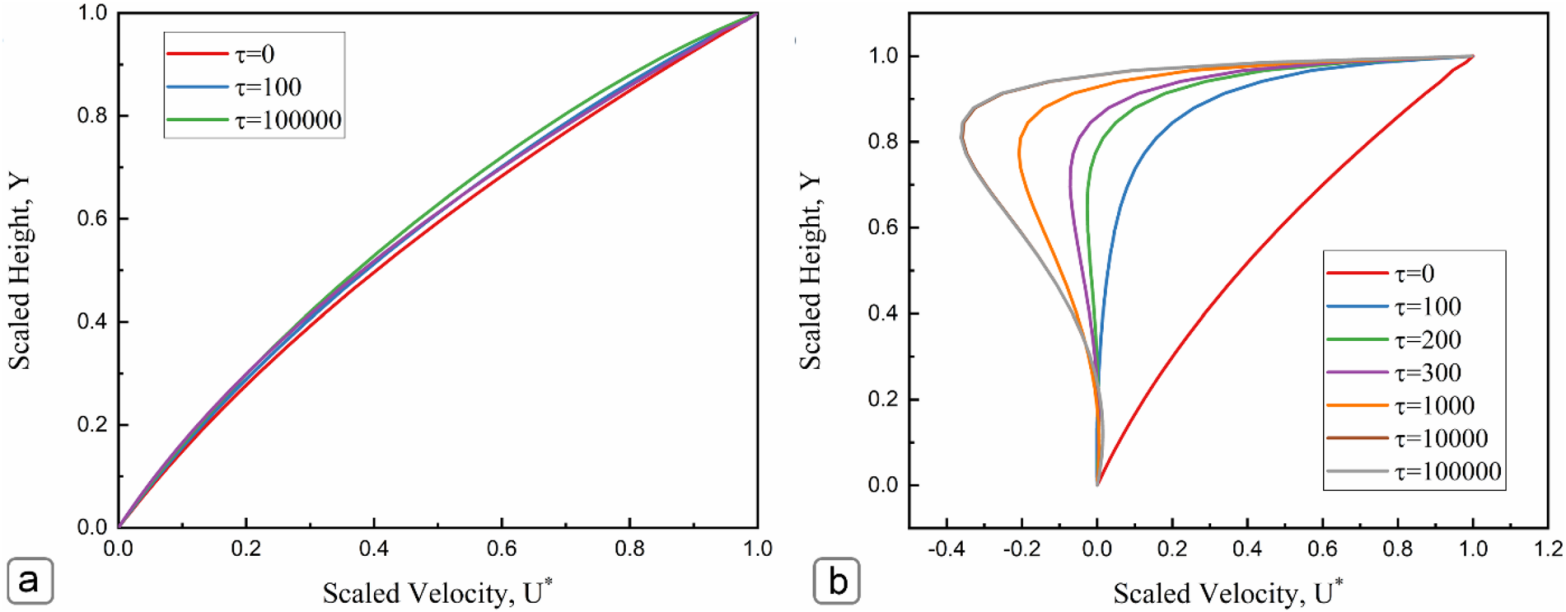
Velocity profile across the non-dimensional channel height for (**a**) Ψ_*s*_ = 1, and (**b**) Ψ_*s*_ = 6. The velocity field was obtained for *κH* = 10*.*

This section presents how the electrokinetic flow develops from an initial quiescent state after applying the moving wall boundary condition. The transient analysis was performed using a diffusion-based time scale $\uptau ={\kappa }^{2}Dt$ where $t$ is the dimensional time. Figure 10 illustrates the velocity profile across the microchannel at low (Ψ_*s*_ = 1) and high (Ψ_*s*_ = 6) surface potentials as time progresses. Based on Fig. 10a, the fluid velocity deviates slightly from the initial profile for low surface potentials, showing a minor impact of the induced electric field on the flow field. This behavior agrees with the analytical velocity profile obtained in Fig. 6. The slight non-linearity of the numerical simulation compared to analytical results is attributed to the effect of entrance/exit effects. In contrast to the analytical analysis where an infinitely long channel was considered with a negligible entrance/exit effect, the velocity distribution in the numerical analysis was obtained for a finite length. Figure 10b presents the variation of scaled velocity across the scaled height of microchannel at the high surface potential of Ψ_*s*_ = 6 and *κH* = 10. At the initial time step (*τ* = 0), there was no streaming potential backflow, and the flow was shear-driven, following the direction of the moving plate. As time progresses (τ = 100), the ions start to move in the axial direction and accumulate at the end reservoir of the microchannel. The accumulation of ions creates a streaming potential that induces an electric field in the opposite direction, which restrained the axial flow. At the time steps τ = 200 to 300, the overall flow starts to stagnate as the streaming potential backflow became almost equal to the shear driven flow in the central part of the channel. The streaming potential flow starts to dominate over the shear-driven flow in time steps of τ = 1000–10,000, and flow reversal occurred near the central part of the channel closer to the charged wall.

**Figure 11:**
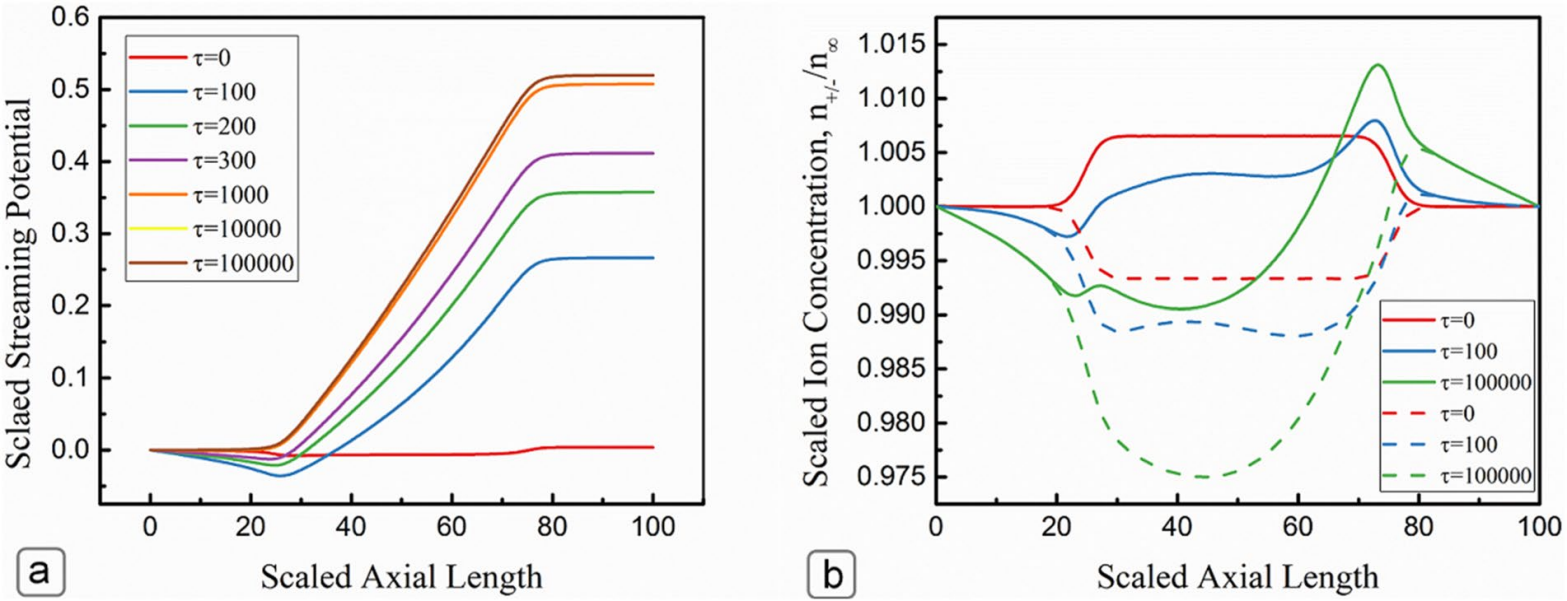
(**a**) Development of streaming potential along the centerline of the channel, (**b**) scaled ion concentration along the centerline of the channel, the dashed line represents the co-ion concentration while the solid lines are for the counterion concentration. *κH* = 10 and Ψ_*S*_ = 1.

Figure 11 illustrates the development of streaming potential and ion concentrations along the centerline of the microchannel. Both the streaming potential and axial ion concentration exhibit similar behavior for both high and low surface potentials; therefore, only the case for low surface potential is presented here. After solving for the initial distributions, the transient Navier–Stokes equations were solved coupled with Poisson–Nernst–Planck equations beginning from an initial no-flow condition. The solid lines in Fig. 11b represent the counter ions concentration while the co-ions were represented by the dashed lines. At the initial state (τ = 0), the electrical potential was almost constant across the channel as there was no axial variation of ions. At this time, the concentration of co-ions was less than the bulk concentration (n/n_∞_ = 1), while counter ions were greater than the bulk concentration. As time progresses, streaming potential developed along the channel. The streaming potential increased rapidly at the first-time step (*τ* = 100) and reached 50% of its steady-state value. As time progressed, the streaming potential increases with a milder rate when it reached its final steady value at *τ* = 1.0 × 10^5^ s. This observation can be attributed to the generation of backward conduction current and streaming potential flow, which limited the ionic buildup near the ends of the microchannel. Figure 11b confirms the justification as it illustrates that the counterions accumulate near the exit of the channel while the co-ions were more depleted towards the entry at a steady state.

**Figure 12:**
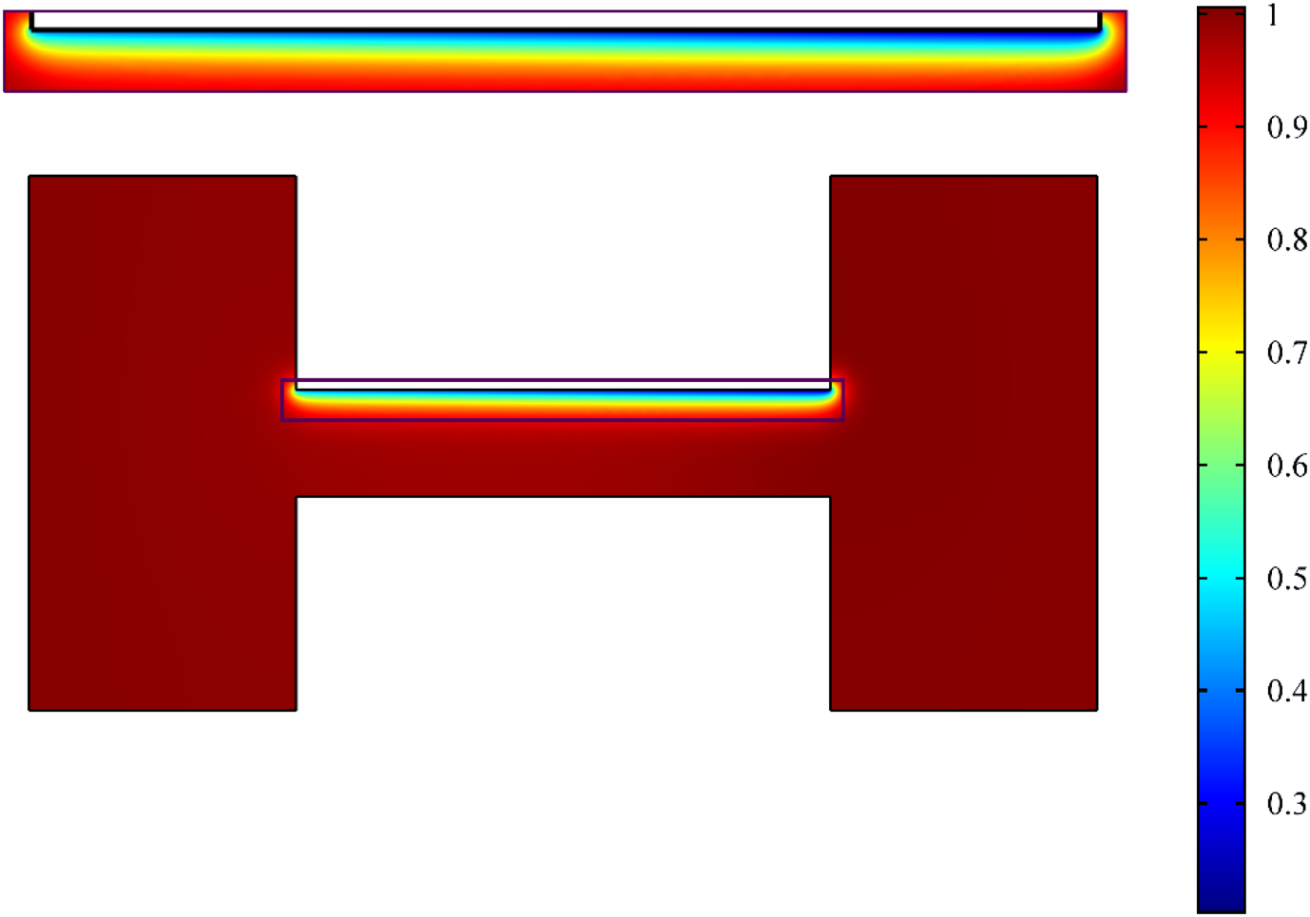
Contour plot of scaled co-ions concentration within the microchannel with a zoomed-in inset of the region near the charged wall highlighting the asymmetric axial variation of the electric double layer. *κH* = 10 and Ψ_S_ = 1.

Figure 12 depicts a contour plot of co-ion concentration in which the co-ion concentration was significantly lower than the bulk concentration due to the formation of the EDL near the wall. The plot also highlights the asymmetry of the shear driven flow in both vertical and horizontal directions. The co-ion concentration near the electric double layer was higher towards the inlet reservoir than to the exit reservoir, which was not similar to what was derived at the centerline due to the overall charge conservation of the solution.

## Conclusion

In the present work, the electrokinetic phenomenon for a shear-driven flow in a charged slit microchannel was analyzed using analytical expressions and numerical simulation. The results show that the surface charge of the moving interface, as well as the thickness of EDL, greatly influenced the flow field within the microchannel. It was obtained that above a threshold, the streaming potential flow could reverse the main shear-driven flow in the region close to the charged wall, resulting in a stationary plane within the flow domain. Furthermore, it was demonstrated that the generation of induced streaming potential within the channel reduced the overall flow rate due to electroviscous effects. Finally, the numerical model showed the transient development of the streaming potential from the electroneutral condition, which provides useful information for a better understanding of electrokinetic flow within a confined media such as a charged microchannels.

## Supplementary information

Supplementary Information.
