# Supplementary material for: Analysis of streaming potential flow and electroviscous effect in a shear-driven charged slit microchannel
Source: Sci Rep. 2020 Oct 27;10:18317. doi: 10.1038/s41598-020-75531-6 (PMC7591916; doi:10.1038/s41598-020-75531-6)
Supplement: Supplementary file 1 — Supplementary Information. [file 41598_2020_75531_MOESM1_ESM.docx]

**Supplementary Information**

**Analysis of streaming potential flow and electroviscous effect in a shear-driven charged slit microchannel**

**Adham Riad, Behnam Khorshidi, Mohtada Sadrzadeh^*^**

Department of Mechanical Engineering, 10-367 Donadeo Innovation Center for Engineering, University of Alberta, Edmonton, AB, Canada, T6G 1H9

**^*^**Corresponding Authors: [sadrzade@ualberta.ca](mailto:sadrzade@ualberta.ca)

The supplementary information includes::

**Section 1:** Analytical derivation for the electrical potential distribution in a shear-driven charged slit microchannel

**Section 2:** Supplementary Figure S1: Comparison of the numerical and analytical predictions of the induced electric field for different surface potentials. (κH=100).

**Section 1: Analytical derivation for the electrical potential distribution in a shear-driven charged slit microchannel**

The analytical derivation for electrical potential distribution within the electric double layer (EDL) in the straight microchannel starts with the Eq. (S1) which is the non-linear Poisson-Boltzmann equation:

$\frac{d^{2}\psi\left( y \right)}{dy^{2}}=\frac{2zen_{\infty}}{\epsilon}\sinh\left( \frac{ze}{K_{B}T}\psi\left( y \right) \right)$ (S1)

Non-dimensionalizing Eq.(S1), using the dimensionless electrical potentials defined as $\Psi\left( y \right)={ze}/{K_{B}T}\psi\left( y \right)$ and$\Psi_{s}={ze}/{K_{B}T}\xi$ leads to Eq.(2):

$\frac{d^{2}\Psi\left( y \right)}{dy^{2}}=\kappa^{2}\sinh\left( \Psi\left( y \right) \right)$ (S2)

The boundary conditions for Eq.(S2) are as follows at $y=0, \Psi\left( y \right)=0$ and at$y=H,{\Psi\left( y \right)=\Psi}_{s}={ze}/{K_{B}T}\xi$

Integrating Eq. (S2) from $y=0$ to an arbitrary y:

$\frac{d\Psi\left( y \right)}{dy}=4\kappa\sinh\left( \frac{\Psi\left( y \right)}{4} \right)\cosh\left( \frac{\Psi\left( y \right)}{4} \right)$ (S3)

Integrating Eq. (S3) from $y=0$ to an arbitrary y:

$\int_{\Psi_{s}}^{\Psi\left( y \right)} \frac{d\Psi\left( y \right)}{sinh\left( \frac{\Psi\left( y \right)}{4} \right)cosh\left( \frac{\Psi\left( y \right)}{4} \right)}=\int_{H}^{y} 4\kappa dy$ (S4)

$\ln\left[ \tanh\left( \frac{\Psi\left( y \right)}{4} \right) \right]-\ln\left[ \tanh\left( \frac{\Psi_{s}}{4} \right) \right]=\kappa(y-H)$ (S5)

Finally rearranging the equation leads to Eq. (S5):

$\Psi\left( y \right)=4 arctanh\left( tanh\left( {\Psi_{s}}/4 \right)exp\left( -\kappa(H-y) \right) \right)$ (S6)

**Section 2: Supplementary figure**

**

**

**Figure S1:** Comparison of the numerical and analytical predictions of the induced electric field for different surface potentials. (*κH*=100).
